# Psychological Challenges in Children With Tracheostomies and Their Families—A Qualitative Study

**DOI:** 10.1111/coa.14249

**Published:** 2024-10-24

**Authors:** Mairi Weir, Haytham Kubba

**Affiliations:** ^1^ Department of Child Health University of Glasgow Glasgow Scotland

**Summary**

- Previous research has shown that paediatric tracheostomy can be a very difficult experience for the whole family. Despite this, as far as we are aware, no paediatric airway service in the United Kingdom includes a dedicated psychologist.
- This study aims to provide information on parents' thoughts regarding the psychological support currently offered in a tertiary paediatric setting.
- This is a qualitative study using in depth interviews to explore themes. The participant group was parents of children under 18 with a current tracheostomy.
- This study demonstrated a high demand for a psychological support service among those under the care of airway services. Parents valued continuity of care and ongoing support throughout the child's journey of care. We did not detect any perceived stigma associated with referral to psychological services.
- Although our study is small, our sample is representative of the targeted cohort and qualitative analysis allowed in depth insight into parents' priorities.

## 1 Introduction

Approximately 1,200 children require a new tracheostomy every year in the United Kingdom [1], and most are in place for more than 2 years, requiring prolonged periods of care at home [2]. The stress and worry associated with tracheostomy care can affect the whole family. Parents often feel an altered sense of their parental role and many suffer poor mental health [3, 4, 5].

As far as we are aware, no paediatric airway service in the UK has a dedicated clinical psychologist who sees all families in the service. In our catchment area, psychological support for children and families is available on referral to the Paediatric Clinical Psychology Service (PCPS) but this is done on a case‐by‐case basis and is not routinely integrated into the airway team. This qualitative study aims to explore parents' thoughts on the psychological support we currently offer at our tertiary paediatric centre by generating and exploring ideas through in‐depth discussions with a small number of test subjects.

## 2 Method

### 2.1 Ethical Considerations

The study was registered with the hospital's clinical governance committee as a service improvement project and was approved by the local information governance officer (known in the UK as the Caldicott Guardian). All participating families gave consent for participation. All information gathered was anonymised unless families specifically requested a referral to the psychology service by giving their contact details.

### 2.2 Participants

The study participants were a convenience sample of outpatient clinic attendees and hospital inpatients seen over 3 months at a tertiary paediatric centre (January–March 2023). The inclusion criterion was any primary caregiver or parent of a child under 18 with a current tracheostomy.

### 2.3 Data Collection and Analysis

**FIGURE 1:**
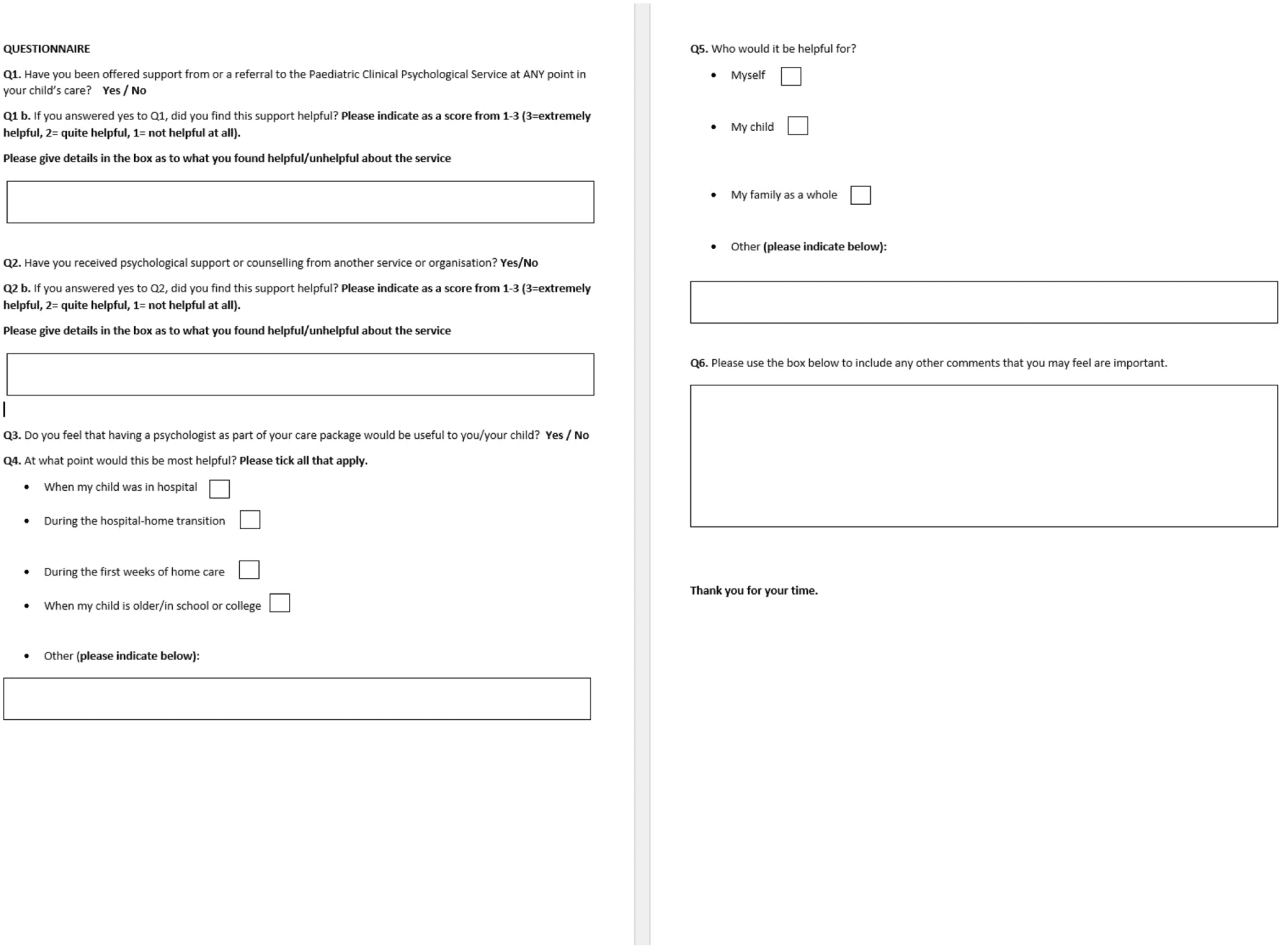
Questionnaire template.

Parents and caregivers were interviewed by one of the study team (MW) who used a short series of questions as a prompt for discussion (see Figure 1). Parents and caregivers were encouraged to give their thoughts freely and discursively. The prompt questions were on their perceptions of the need for psychological support, whether they had received help from PCPS or other psychology services, and how they felt support should be tailored to best support them. Participants were asked to rate any psychological support they received. Participants were invited to provide their name and contact details only if they wished to be referred to the PCPS.

**FIGURE 2:**
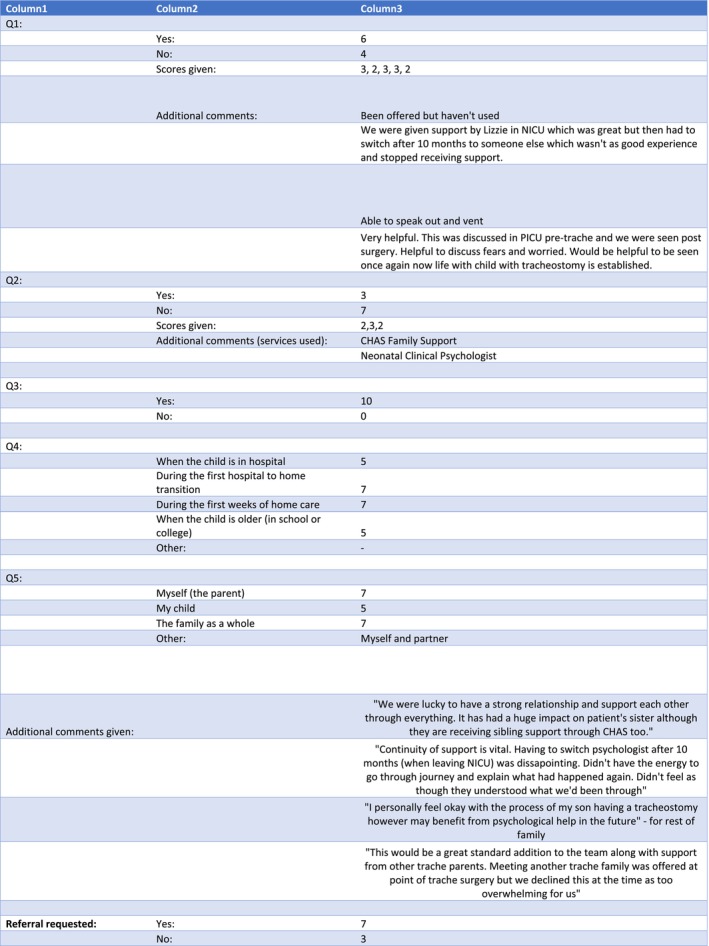
Questionnaire responses.

Only a small number of quantitative analyses were performed: the proportion of participants requesting referral to PCPS, the proportion who had already received psychological support and their rating of its usefulness. The majority of study analysis was done qualitatively, based on Grounded Theory. This is a descriptive process where participant responses are grouped into subject areas and themes, and results are usually presented with illustrative quotations. The number of subjects in qualitative studies is usually small (single figures are common) and the idea is to generate ideas rather than test hypotheses and produce *p* values (Figure 2).

### 2.4 Guidelines

The SQUIRE guidance for quality improvement projects was used when designing and writing this study.

## 3 Results

### 3.1 Study Participants

Ten children with tracheostomy were identified and families were recruited to the study. All study participants were parents. The children were aged 2 months–4 years (median 13 months) and 5 were boys. The most common indications for a tracheostomy were airway obstruction as part of a congenital syndrome (CHARGE, Stickler, Pierre Robin, Apert) and tracheomalacia. The tracheostomy had been in place for between 1 and 34 months at the time of interview (median 11 months). None of the children had suffered an unusually complicated or difficult recovery after the tracheostomy procedure. In terms of age, sex and indication for tracheostomy, the children were typical of those in our tracheostomy cohort [2], which usually numbers between 50 and 70 patients at any time.

Six of the 10 participants said that they had received a previous referral to PCPS. Of those who received support from PCPS, four participants rated it ‘very helpful’ and two rated it ‘quite helpful’. Of the three who received support from other sources (Children's Hospice Family Support and a psychologist on the neonatal unit), two rated them ‘quite helpful’ and one rated them ‘very helpful’. All 10 participants agreed that a psychologist would be a useful routine addition to their team of clinicians. Seven of the 10 participants requested a referral to PCPS after completing the study.

For the qualitative part of the study, four major themes were identified in the responses. These were ‘freedom to express worries’, ‘continuity of care’, ‘extended period of support’ and ‘family dynamics’.

### 3.2 Freedom to Express Worries

Parents mentioned how important it is to be able to freely express their thoughts: ‘Helpful to discuss fears and worries’. Parents frequently responded that they would like the ability to ‘speak out and vent’.

### 3.3 Continuity of Care

Many parents stressed the importance of continuity of care. They wanted to have just one psychologist and felt distressed when having to change to a different one. One participant said, ‘Continuity of support is vital. Having to switch psychologists when leaving NICU was disappointing. Didn't have the energy to go through the journey and explain what had happened again’.

### 3.4 Extended Period of Support

The importance of support beyond the time of the tracheostomy insertion was stressed by a number of participants. One parent said, ‘It would be helpful to be seen [by PCPS] once again now life with a child with a tracheostomy is established’. With regard to when support from PCPS would be most useful in the family's life, frequent answers were, ‘during the first hospital to home transition’, ‘during the first weeks of home care’, ‘when my child is in hospital’ and ‘when my child is older and in school or college’. One response was, ‘This would be a great standard addition to the team … was offered at the point of tracheostomy surgery but we declined this at the time as too overwhelming for us’.

### 3.5 Family Dynamics

Some parents expressed how the strength of their relationship with each other, and their other children, influenced their experiences with a tracheostomy. One typical response was, ‘We were lucky to have a strong relationship and support each other through everything. It has had a huge impact on the patient's sister although they are receiving sibling support through [Children's Hospice Family Support] too’. With regard to who the study participants felt would benefit from psychological support, frequent answers included ‘myself’, ‘my family as a whole’ and ‘my child’. One parent said, ‘I personally feel okay with the process of my son having a tracheostomy however may benefit from psychological help in the future for the rest of my family’.

## 4 Discussion

Parents of children with tracheostomy are well‐trained in clinical aspects of care and most rapidly become experts in their child's condition [6]. Some parents consider their child's quality of life to be higher than their own [7] and lower perceived quality of life may have a negative impact on a parent's caregiving abilities. Some parents struggle to participate in their child's tracheostomy care due to feeling overwhelmed [8]. Psychological support can be invaluable in improving the long‐term outcomes for both patient and their family. Previous studies have suggested that having a member of staff on hand who is familiar with the family can be invaluable [9]. Psychological support needs change over time, with parents wanting help in the early stages, while children may benefit most when they are older, especially during important times of transition such as starting school [10].

The main value of qualitative research is in identifying areas that are important to patients but which might be surprising to clinicians. Our biggest surprise was the lack of any perceived stigma in accessing psychological services. We expected this to be a major barrier to families accessing care but we were pleasantly surprised to see no sign of reticence on the part of parents in seeking psychological support. On the contrary, 70% actively requested referrals.

Our study is small, but that is often the way with qualitative studies, where in‐depth analysis of response themes is more important than large study numbers. Our sample is representative of our tracheostomy cohort overall and the responses were consistent from family to family. The qualitative element of the study allowed us insights into parents' priorities, particularly the importance of long‐term continuity in care. The main outcome is the strong demand from parents for integrated psychological support as a routine part of our airway service. Further quantitative work could focus on assessing the benefit of psychological support, and whether it is more beneficial for particular families such as those whose child had a more complicated course, or where the tracheostomy is in place for a longer time.

## Author Contributions

H.K. designed the work. M.W. acquired and analysed data. M.W./H.K. drafted, revised and approved the manuscript. H.K. acts as guarantor for the integrity of all aspects of the work.

## Ethics Statement

This study has been conducted in line with the ethical considerations outlined by the Caldicott guidelines. All participants gave informed consent and were given the right to withdraw at any time. All data was anonymised.

## Conflicts of Interest

The authors declare no conflicts of interest.

### Peer Review

The peer review history for this article is available at https://www.webofscience.com/api/gateway/wos/peer‐review/10.1111/coa.14249.

## Data Availability

The data that support the findings of this study are available from the corresponding author upon reasonable request.
